# Report of Deaths in the City of Buffalo for the Month of December, 1861

**Published:** 1862-02

**Authors:** J. Whittaker

**Affiliations:** Health Physician


*Report of Deaths in the City of Buffalo for the month of December*, 1861

Accidents, 2; Accident by drowning, 4; Anæmia, 1; Apoplexy, 2; Bronchitis, 1; Consumption, 7; Convulsions, 10; Croup, 7; Cyanosis, 1; Debility, 2; Delirium Tremens, 5; Diarrhœa, 5; Disease of the Brain, 1; Disease of the Heart, 3; Disease of the Lungs, 8; Diphtheria, 5; Dropsy, 2; Dropsy Abdominal, 1; Dropsy of the Brain, 4; Dysentery, 1; Epilepsy, 1; Fever, Scarlet 11; Inflammation of Brain, 4; Inflammation of Lungs, 5; Inflammation of Lungs, Typhoid, 1; Inflammation of Pericardium, 1; Inflammation of Peritoneum, 1; Intemperance, 2; Intussusception, 1; Marasmus, 1; Old Age, 4; Premature Birth, 1; Scrofula, 3; Small-pox, 1; Ulcer, 1; Whooping Cough, 4; Unknown 3. Under one year of age, 28; between one and twenty, 50; between twenty and fifty, 17; over fifty, 21. Still-born, 1. Total, 118.

J. WHITTAKER, *Health Physician*.

